# Papilliferous Keratoameloblastoma: An Extremely Rare Case Report

**DOI:** 10.1155/2013/706128

**Published:** 2013-06-03

**Authors:** Neeta Mohanty, Varun Rastogi, Satya Ranjan Misra, Susant Mohanty

**Affiliations:** ^1^Department of Oral and Maxillofacial Pathology, Institute of Dental Sciences, Bhubaneswar, Odisha 751003, India; ^2^Department of Oral and Maxillofacial Pathology, Kalka Dental College, Meerut, Uttar Pradesh 250 006, India; ^3^Department of Oral Medicine and Radiology, Institute of Dental Sciences, Bhubaneswar, Odisha 751003, India; ^4^Department of Paediatric Dentistry, Institute of Dental Sciences, Bhubaneswar, Odisha 751003, India

## Abstract

Odontogenic tumors develop in the jaw bones from the odontogenic tissue-oral epithelium in tooth germ, enamel organ, dental papilla, reduced enamel epithelium, remnants of Hertwig's root sheath or dental lamina, and so forth. Hence, a bewildering variety of tumors are encountered in the maxilla and mandible. Ameloblastoma is the second most common odontogenic neoplasm after odontomes, and it has numerous clinical and histologic variants. We report a very rare histologic variant: the papilliferous keratoameloblastoma which is the fifth reported case in the English literature.

## 1. Introduction 

Ameloblastoma is a true neoplasm of enamel organ-type, tissue which does not undergo differentiation to the point of enamel formation. It has been described very aptly by Robinson as being a tumor that is usually “unicentric, nonfunctional, intermittent in growth, anatomically benign, and clinically persistent” [1].

Ameloblastoma is the most common odontogenic epithelial tumor of the jaw bones [2], and it is characterized by a substantial number of histologic subtypes [3]. Recognition of the various histomorphic patterns is thus only of diagnostic significance and is especially important for histopathologists, despite the fact that they are generally considered to be locally aggressive and destructive, exhibiting various rates of recurrences.

Variations of ameloblastoma that exhibit keratinization in their parenchyma are the Acanthomatous ameloblastoma, Keratoameloblastoma (KA), and Papilliferous Keratoameloblastoma (PKA). Of these, Keratoameloblastoma and Papilliferous Keratoameloblastoma are extremely rare tumors, and despite the similarity of the names, Keratoameloblastoma and Papilliferous Keratoameloblastoma are distinct morphologically [4]. 

Pindborg [5] first proposed the term “Keratoameloblastoma” for use as a diagnostic entity. In his 1970 report, Pindborg described a histologic variant of ameloblastoma, which he termed Papilliferous Keratoameloblastoma. Subsequently, 3 additional cases of ameloblastoma with a Papilliferous component were reported in 1991 [3], 1994 [6], and 2002 [7], bringing the number of case reports of PKA in the English Language literature to four. We report a case of Papilliferous Keratoameloblastoma which is the 5th case to document in the English literature. 

## 2. Case Report

A 46-year male patient reported to the dental hospital with the complaint of swelling in the right side of the lower jaw since 1 year. The swelling progressed slowly to the present side with no history of toothache, and the past medical history is noncontributory.

**Figure 1:**
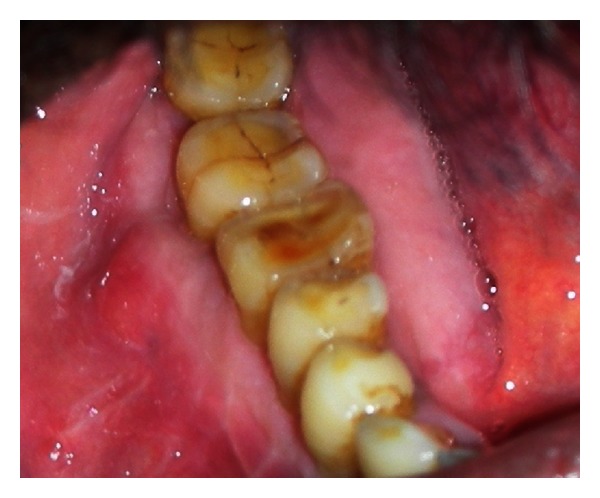
Intraoral swelling with bicortical expansion extending from 44 to 48 region.

On examination, a single localized swelling was seen in the right side body of the mandible, ovoid in shape, about 2 cm in greatest diameter, nontender, bony hard in consistency, and fixed to the underlying bone. Intraorally, a swelling was seen having bicortical expansion extending from 44 to 48 region (Figure 1). It was nontender and firm in consistency, and it obliterates the buccal vestibule. A provisional diagnosis of benign bony neoplasm was made, and the patient subjected to radiologic investigations.

**Figure 2:**
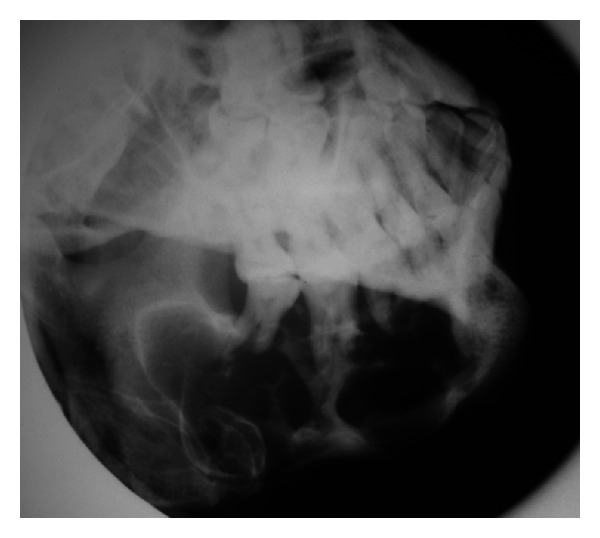
Lateral oblique projection of the body of the mandible showing a multilocular radiolucency, extending from first premolar to the angle of the mandible.

**Figure 3:**
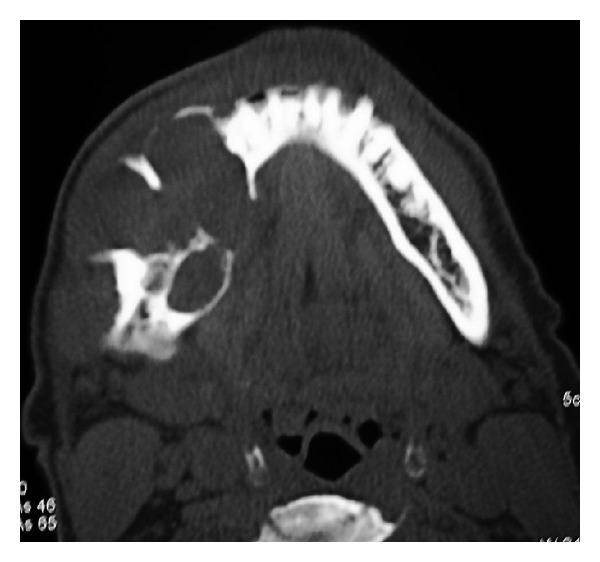
Axial view CT scan showing bicortical expansion with perforation of the buccal and lingual cortices on the right side.

**Figure 4:**
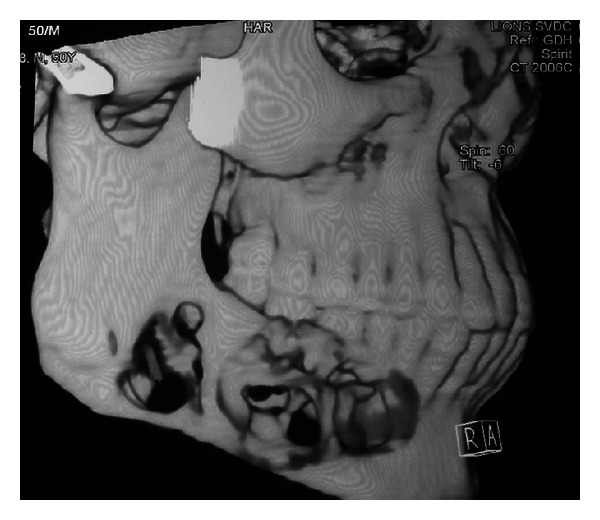
3D reformatted CT scan showing perforation of the mandible from 44 to angle region on right side.

Periapical, occlusal, and lateral oblique projections of the mandible revealed a multilocular radiolucency, extending from the first premolar to the angle of the mandible (Figure 2); there is bicortical expansion with perforation of both the buccal and lingual cortex. There is root resorption of the involved teeth. Computer Tomography scans also revealed bicortical expansion and cortical perforation (Figures 3 and 4). 

**Figure 5:**
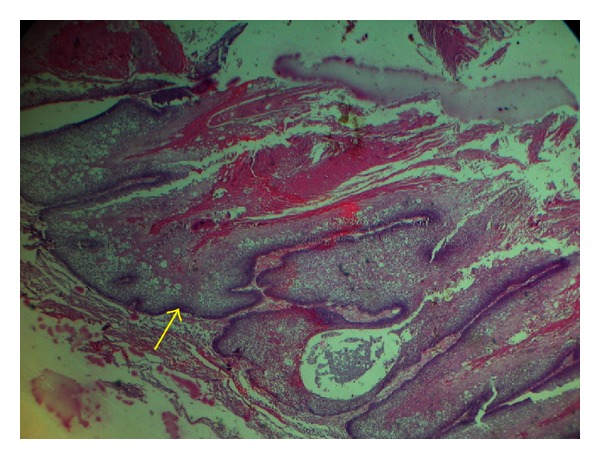
H & E section (10X) showing cystic spaces filled with the necrotic debris and lined by papillary (Yellow arrow) keratin lined infolding of odontogenic epithelium resembling ameloblastoma with connective tissue cores.

**Figure 6:**
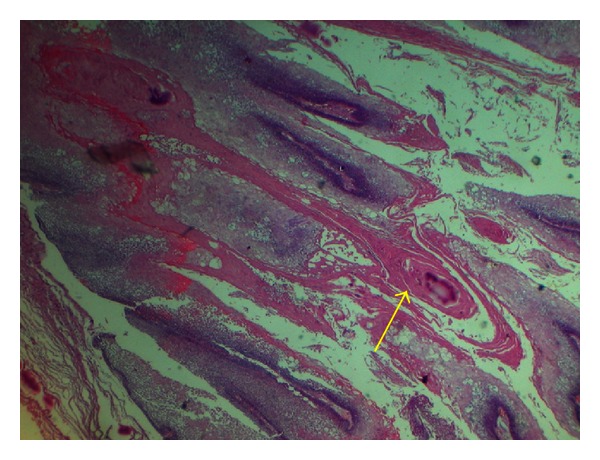
H & E section (10X) showing cystic spaces lined by papillary keratin lined infolding (Yellow arrow) of odontogenic epithelium.

**Figure 7:**
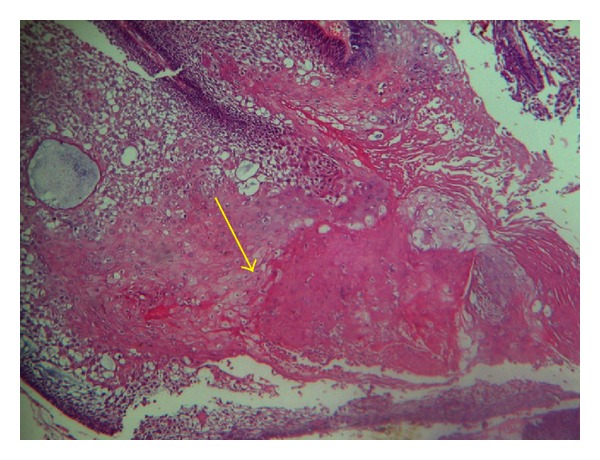
Cystic spaces showing squamous metaplasia (Yellow arrow) and presence of keratin squames (H & E 10X).

**Figure 8:**
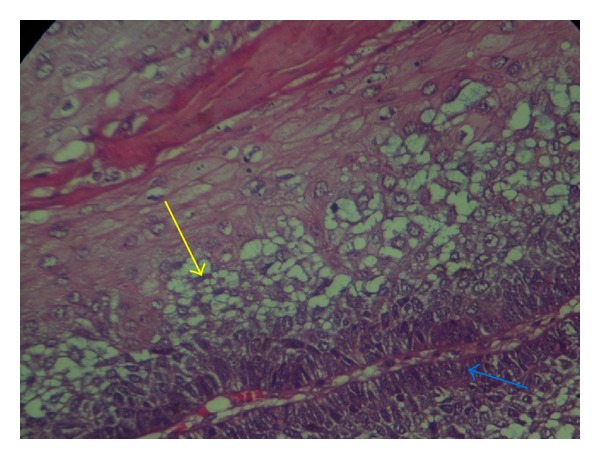
Odontogenic epithelium consisting of loosely arranged polygonal/angular cells resembling stellate reticulum (Yellow arrow) of the enamel organ and basal layer of tall columnar ameloblast-like cells (Blue arrow) showing palisading and reversal of polarity (H & E 40X).

**Figure 9:**
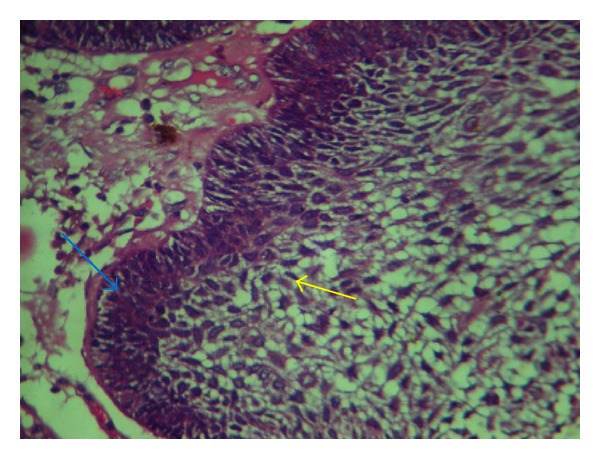
Loosely arranged polygonal/angular cells resembling stellate reticulum (Yellow arrow) of the enamel organ and basal layer of tall columnar ameloblast-like cells (Blue arrow) showing palisading and reversal of polarity (H & E 40X).

An incisional biopsy of the lesion was done, and, microscopically, the lesion consisted of cystic spaces filled with the necrotic debris and lined by papillary keratin lined infolding of odontogenic epithelium resembling ameloblastoma with connective tissue cores (Figures 5 and 6). The cystic spaces also showed squamous metaplasia and presence of keratin squames (Figure 7). The odontogenic epithelium consisted of loosely arranged polygonal or angular cells resembling stellate reticulum of the enamel organ (Figure 8) and basal layer of tall columnar ameloblast-like cells showing palisading and reversal of polarity (Figures 8 and 9). Correlating the histopathological features, a definitive diagnosis of Papilliferous keratoameloblastoma of the mandible was made, and the patient was referred to the oral surgery department for management.

## 3. Discussion

Ameloblastomas undergo different forms of metaplasia and are therefore highly polymorphic benign odontogenic tumors, giving rise to the histologic variants like acanthomatous, granular cell, desmoplastic, basal cell, keratoameloblastoma, and clear-cell ameloblastoma. The cause or the stimulus for these metaplasias is unknown; however, it is generally attributed to the multipotentiality of odontogenic epithelium [8, 9].

Histopathologically, ameloblastoma recapitulates the morphology of the prematrix stage of the enamel organ and may assume a variety of histological configurations. Clinically, no behavioural difference has been observed among histological and cytological variants.

Papilliferous keratoameloblastoma and Keratoameloblastoma are extremely rare variants of ameloblastoma [10]. Papilliferous keratoameloblastoma was first described as a subtype of ameloblastoma by Pindborg and Weinmann [11]. In 1970, Pindborg [5] produced a radiograph and three photomicrographs of an unusual type of ameloblastoma consisting partly of keratinizing cysts and partly of tumor islands with Papilliferous appearance, and the term Papilliferous keratoameloblastoma was then suggested [12]. Later, Altini et al. [3] described a similar tumor that differed from the one described by Pindborg in that it did not show the Papilliferous epithelium nor the extensive necrosis and debris in the follicles.

Since only one of the four cases showed convincing evidence of ameloblastoma, there is minimal evidence that they are ameloblastoma and may represent a separate entity. This finding is consistent with our case which showed histopathological features of ameloblastoma. 

The Papilliferous nature of the epithelium seems to have occurred as result of intercellular adherence and different rates of necrosis of individual cells [13]. The necrotic cells separate from the remainder of the epithelium resulting in the formation of numerous pseudopapillary structures which project into the lumina of the cystic follicles.

Keratoameloblastoma, Papilliferous Keratoameloblastoma, or a possible hybrid lesion of the two is an extremely rare neoplasm, and an accurate evaluation of the clinical spectrum, radiology, and behavioural potential must await further case accrual.
